# Endothelial Ca^2+^ Signaling and the Resistance to Anticancer Treatments: Partners in Crime

**DOI:** 10.3390/ijms19010217

**Published:** 2018-01-11

**Authors:** Francesco Moccia

**Affiliations:** Laboratory of General Physiology, Department of Biology and Biotechnology “L. Spallanzani”, University of Pavia, I-27100 Pavia, Italy; francesco.moccia@unipv.it; Tel.: +39-382-39-0382-987527

**Keywords:** Ca^2+^ signaling, tumor, endothelial cells, endothelial progenitor cells, endothelial colony forming cells, anticancer therapies, VEGF, resistance to apoptosis

## Abstract

Intracellular Ca^2+^ signaling drives angiogenesis and vasculogenesis by stimulating proliferation, migration, and tube formation in both vascular endothelial cells and endothelial colony forming cells (ECFCs), which represent the only endothelial precursor truly belonging to the endothelial phenotype. In addition, local Ca^2+^ signals at the endoplasmic reticulum (ER)–mitochondria interface regulate endothelial cell fate by stimulating survival or apoptosis depending on the extent of the mitochondrial Ca^2+^ increase. The present article aims at describing how remodeling of the endothelial Ca^2+^ toolkit contributes to establish intrinsic or acquired resistance to standard anti-cancer therapies. The endothelial Ca^2+^ toolkit undergoes a major alteration in tumor endothelial cells and tumor-associated ECFCs. These include changes in TRPV4 expression and increase in the expression of P2X7 receptors, Piezo2, Stim1, Orai1, TRPC1, TRPC5, Connexin 40 and dysregulation of the ER Ca^2+^ handling machinery. Additionally, remodeling of the endothelial Ca^2+^ toolkit could involve nicotinic acetylcholine receptors, gasotransmitters-gated channels, two-pore channels and Na^+^/H^+^ exchanger. Targeting the endothelial Ca^2+^ toolkit could represent an alternative adjuvant therapy to circumvent patients’ resistance to current anti-cancer treatments.

## 1. Introduction

An increase in intracellular Ca^2+^ concentration ([Ca^2+^]_i_) has long been known to play a crucial role in angiogenesis and arterial remodeling [1,2,3,4,5]. Accordingly, growth factors and cytokines, such as vascular endothelial growth factor (VEGF), epidermal growth factor (EGF), basic fibroblast growth factor (bFGF), insulin-like growth factor-1 (IGF-1), angiopoietin and stromal derived factor-1α (SDF-1α), trigger robust Ca^2+^ signals in vascular endothelial cells [6,7,8,9,10,11,12], which recruit a number of downstream Ca^2+^-dependent pro-angiogenic decoders. These include, but are not limited to, the transcription factors, Nuclear factor of activated T-cells (NFAT), Nuclear factor-kappaB (NF-κB) and cAMP responsive element binding protein (CREB) [8,13,14], myosin light chain kinase (MLCK) and myosin 2 [8,15], endothelial nitric oxide synthase (eNOS) [16,17], extracellular signal–regulated kinases ½ (ERK 1/2) [18,19] and Akt [19,20]. Not surprisingly, therefore, subsequent studies clearly revealed that endothelial Ca^2+^ signals may also drive tumor angiogenesis, growth and metastasis [3,21,22,23,24]. However, the process of tumor vascularization is far more complex than originally envisaged [25]. Accordingly, the angiogenic switch, which is the initial step in the multistep process that ensures cancer cells with an adequate supply of oxygen and nutrients and provides them with an escape route to enter peripheral circulation, is triggered by the recruitment of bone marrow-derived endothelial progenitor cells (EPCs), according to a process termed vasculogenesis [26,27,28]. Similar to mature endothelial cells, EPCs require an increase in [Ca^2+^]_i_ to proliferate, assembly into capillary-like tubular networks in vitro and form patent neovessels in vivo [29,30,31]. Of note, intracellular Ca^2+^ signals finely regulate proliferation and in vitro tubulogenesis also in tumor-derived EPCs (T-EPCs) [23,32,33]. An established tenet of neoplastic transformation is the remodeling of the Ca^2+^ machinery in malignant cells, which contributes to the distinct hallmarks of cancer described by Hanahan and Weinberg [34,35,36]. Tumor endothelial cells (T-ECs) and T-EPCs do not derive from the malignant clone, but they display a dramatic dysregulation of their Ca^2+^ signaling toolkit [29,32,37]. The present article surveys the most recent updates on the remodeling of endothelial Ca^2+^ signals during tumor vascularization. In particular, it has been outlined which Ca^2+^-permeable channels and Ca^2+^-transporting systems are up- or down-regulated in T-ECs and T-EPCs and how they impact on neovessel formation and/or apoptosis resistance in the presence of anti-cancer drugs. Finally, the hypothesis that the remodeling of endothelial Ca^2+^ signals may be deeply involved in tumor resistance to standard therapeutic treatments, including chemotherapy, radiotherapy and anti-angiogenic therapy is widely discussed.

## 2. Ca^2+^ Signaling in Normal Endothelial Cells: A Brief Introduction

The resting [Ca^2+^]_i_ in vascular endothelial cells is set at around 100–200 nM by the concerted interaction of three Ca^2+^-transporting systems, which extrude Ca^2+^ across the plasma membrane, such as the Plasma-Membrane Ca^2+^-ATPase and the Na^+^/Ca^2+^ exchanger (NCX), or sequester cytosolic Ca^2+^ into the endoplasmic reticulum (ER), the largest intracellular Ca^2+^ reservoir [2,38,39,40], such as the SarcoEndoplasmic Reticulum Ca^2+^-ATPase (SERCA). Endothelial cells lie at the interface between the vascular wall and the underlying tissue; therefore, they are continuously exposed to a myriad of low levels soluble factors, including growth factors, hormones and transmitters, which may induce highly localized events of inositol-1,4,5-trisphosphate (InsP_3_)-dependent Ca^2+^ release from the ER even in the absence of global cytosolic elevations in [Ca^2+^]_i_ [41,42,43,44,45]. These spontaneous InsP_3_-dependent Ca^2+^ microdomains are redirected towards the mitochondrial matrix through the direct physical association specific components of the outer mitochondrial membrane (OMM) with specialized ER regions, which are known as mitochondrial-associated membranes (MAMs) [46]. This constitutive ER-to-mitochondria Ca^2+^ shuttle drives cellular bioenergetics by activating intramitochondrial Ca^2+^-dependent dehydrogenases, such as pyruvate dehydrogenase, NAD-isocitrate dehydrogenase and oxoglutarate dehydrogenase [47,48,49]. This pro-survival Ca^2+^ transfer may be switched into a pro-death Ca^2+^ signal by various apoptotic stimuli [46,47,50]. For instance, hydrogen peroxide (H_2_O_2_), menadione, resveratrol, ceramide, and etoposide boost the InsP_3_-dependent ER-to-mitochondria Ca^2+^ communication, thereby causing a massive increase in mitochondrial Ca^2+^ concentration ([Ca^2+^]_mit_), which ultimately results in the opening of mitochondrial permeability transition pore and in the release of pro-apoptotic factors into the cytosol [46,51,52,53]. The hypoxic microenvironment of a growing tumor may then trigger an oxygen (O_2_)-sensitive transcriptional program in tumor cells by activating two basic helix-loop-helix transcription factors, i.e., the hypoxia-inducible factors HIF-1 and HIF-2, which drive the expression of a myriad of growth factors and cytokines [54]. These include, but are not limited to, VEGF, EGF, bFGF, IGF-1, angiopoietin and SDF-1α [27,54], which are liberated into peripheral circulation according to a concentration gradient, which delivers a strong pro-angiogenic signal to vascular endothelial cells residing in close proximity to the primary tumor site [3,33]. Growth factors bind to their specific tyrosine kinase receptors (TKRs), such as VEGFR-2 (KDR/Flk-1), EGFR (ErbB-1), and IGF-1R, thereby stimulating phospholipase Cγ (PLCγ) to cleave phosphatidylinositol 4,5-bisphosphate (PIP_2_) into the two intracellular second messengers, InsP_3_ and diacylglycerol (DAG) [1,2,55]. The following increase in cytosolic InsP_3_ levels further stimulates ER-dependent Ca^2+^ release through InsP_3_ receptors (InsP_3_Rs), which can be amplified by the recruitment of adjoining ryanodine receptors (RyRs) through the process of Ca^2+^-induced Ca^2+^ release (CICR) [1,2]. The following drop in ER Ca^2+^ concentration ([Ca^2+^]_ER_) is detected by Stromal Interaction Molecule 1 (Stim1), a sensor of ER Ca^2+^ levels, which is prompted to aggregate into oligomers and relocate towards ER-plasma membrane junctions, known as puncta and positioned in close vicinity to the plasma membrane (10–20 nm). Herein, Stim1 interacts with and gates the Ca^2+^-permeable channel, Orai1, thereby triggering the so-called store-operated Ca^2+^ entry (SOCE), the most important Ca^2+^ entry route in endothelial cells [42,56,57,58,59]. In addition, Stim1 may recruit additional Ca^2+^-permeable channels, which belong to the Canonical Transient Receptor Potential (TRPC) sub-family [2,59]. Accordingly, the TRP superfamily of cation channels comprises 28 members, subdivided into six sub-families: TRPC, TRPV (Vanilloid), TRPM (Melastatin), TRPP (Polycystin), TRPML (Mucolipin) and TRPA (Ankyrin) based on the homology of their amino acid sequences [60]. More specifically, endothelial SOCE could involve TRPC1 and TRPC4, which are recruited by Stim1 into a supermolecular heteromeric complex [61], whose Ca^2+^ selectivity is determined by Orai1 [62,63]. Moreover, TRPC3 and TRPC6 may mediate DAG-induced Ca^2+^ entry in several types of endothelial cells [64,65]. This toolkit of Ca^2+^ release/entry channels may be differently exploited by growth factors to stimulate angiogenesis by eliciting diverse patterns of Ca^2+^ signals depending on the vascular bed. For instance, VEGF triggers a biphasic increase in [Ca^2+^]_i_ in human umbilical vein endothelial cells (HUVECs), which consists in an initial InsP_3_-dependent Ca^2+^ peak followed by a plateau phase of intermediate amplitude due to SOCE activation [57,58]. Likewise, InsP_3_ and SOCE shape VEGF- and EGF-induced intracellular Ca^2+^ oscillations in sheep uterine artery endothelial cells [66] and in rat microvascular endothelial cells (CMECs) [7], respectively. VEGF-induced Ca^2+^ influx in HUVECs may, however, be sustained by TRPC3, which causes Na^+^ accumulation beneath the plasma membrane and stimulates the forward (i.e., Ca^2+^ entry) mode of NCX [18]. Moreover, the DAG-gated channel, TRPC6, underlies the monotonic increase in [Ca^2+^]_i_ induced by VEGF in human dermal microvascular endothelial cells (HDMECs) [67]. Finally, TRPC1 is engaged by bFGF to mediate Ca^2+^ entry in HDMECs [68]. These data have been recently confirmed by directly monitoring angiogenesis in developing zebrafish; this model showed that VEGF stimulated biphasic Ca^2+^ signals to drive migration in stalk cells and intracellular Ca^2+^ oscillations to promote proliferation in tip cells [8]. Besides growth factors-activated channels, vascular endothelial cells dispose of a larger toolkit of plasmalemmal Ca^2+^-permeable channels that can be recruited by a multitude of chemical and physical stimuli [2]. For instance, endothelial Ca^2+^ entry may be mediated by additional intracellular second messengers, such as arachidonic acid (AA) and AA metabolites, i.e., epoxyeicosatrienoic acids (EETs) and 2-arachidonoylglycerol, which activate TRPV4 [69,70]; NO, which gates TRPC5 [71]; adenosine 5′-diphosphoribose (ADPR) and low micromolorar doses of H_2_O_2_, which converge on TRPM2 activation [72]; and cyclic nucleotides [73]. Moreover, vascular endothelial cells are endowed with several Ca^2+^-permeable ionotropic receptors, including ATP-sensitive P_2X_ receptors [74], acetylcholine-sensitive nicotinic receptors [75], and *N-*methyl-d-aspartate (NMDA) receptors [76]. Finally, mechanical stimuli (e.g., laminar shear stress, pulsatile stretch, and changes in the local osmotic pressure) elicit Ca^2+^ influx by recruiting a variety of mechano-sensitive channels, such as TRPP2 [77], heteromeric TRPC1-TRPP2 [78], TRPV4 [79], TRPC1-TRPP2-TRPV4 [80], and Piezo1 [81]. Recently, the Ca^2+^ toolkit has also been explored in human EPCs [29]; most of the work has been carried out in endothelial colony forming cells (ECFCs), which represent the only EPC subset truly belonging to the endothelial, rather than the myeloid, lineage [82]. VEGF triggers pro-angiogenic intracellular Ca^2+^ oscillations in ECFCs by triggering the interaction between InsP_3_-dependent Ca^2+^ release and SOCE, which is mediated by Stim1, Orai1 and TRPC1 [83,84]. Conversely, RyRs and the DAG-sensitive channels, TRPC3 and TRPC6, are absent and do not contribute to Ca^2+^ signaling [84,85,86]. Of note, AA may promote proliferation by directly activating TRPV4 and inducing NO release in the presence of extracellular growth factors and cytokines [86]. Finally, the InsP_3_-dependent ER-to-mitochondria Ca^2+^ shuttle is at work and finely regulates the sensitivity to apoptotic stimuli in ECFCs, too [87].

**Figure 1:**
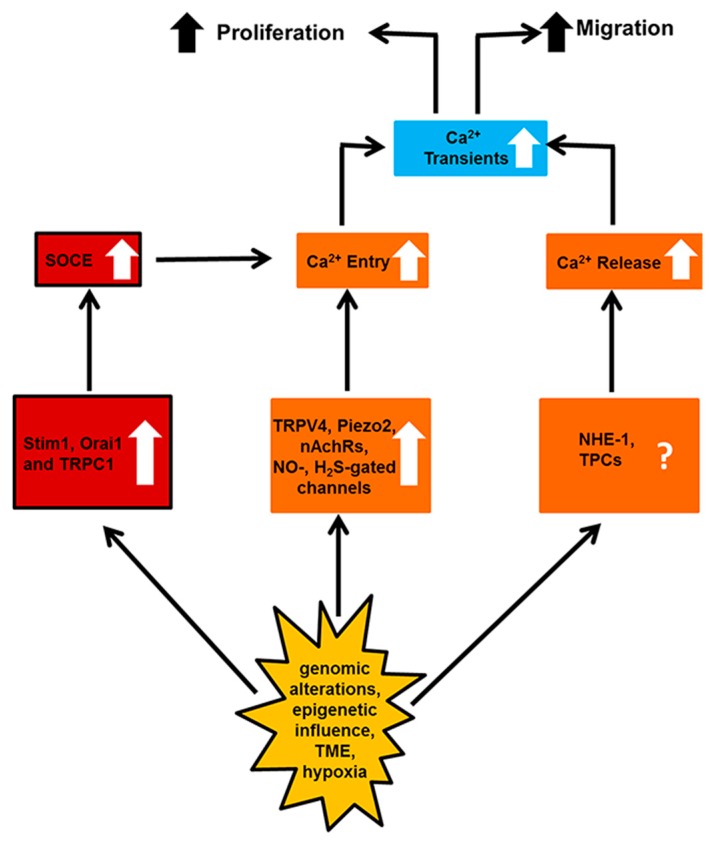
The endothelial Ca^2+^ transportome is remodeled to sustain tumor vascularization. The sequence of events is illustrated by the black arrows. Upward arrows indicate the over-expression of a specific Ca^2+^-permeable channel or transporter and the stimulation of a precise cellular process. See the text for further details.

**Figure 2:**
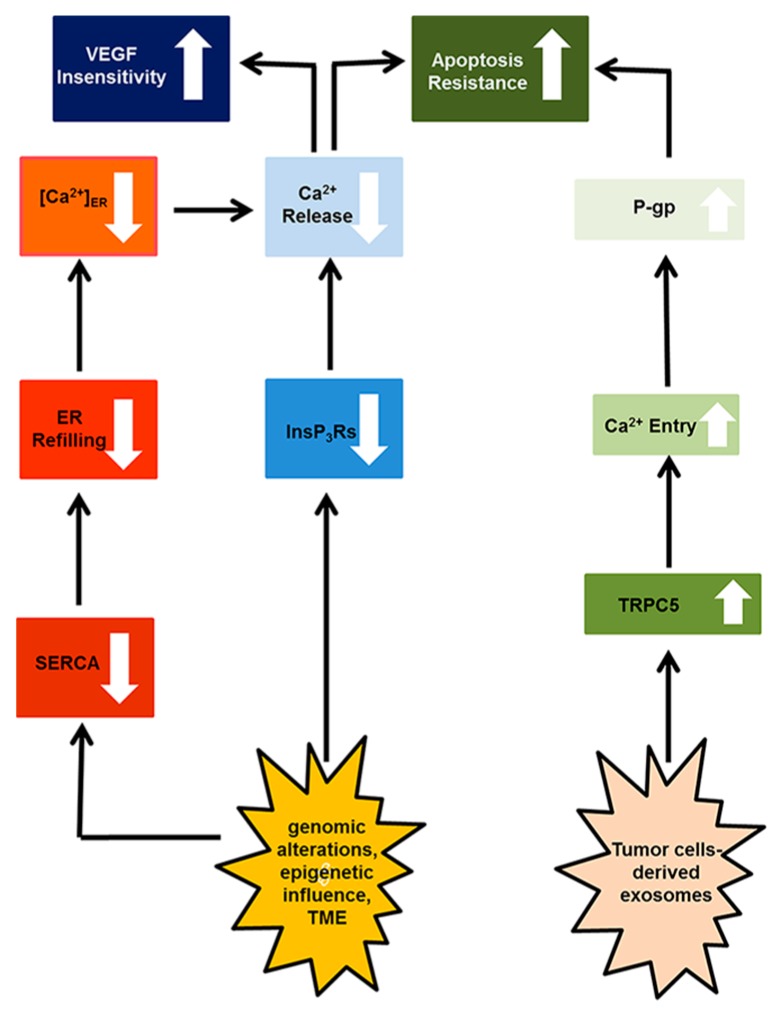
The endothelial Ca^2+^ transportome is remodeled to promote tumor endothelial cell resistance to apoptosis. The sequence of events is illustrated by the black arrows. Downward arrows indicate the down-regulation of a specific Ca^2+^-permeable channel/transporter or of a precise cellular process. Upward arrows indicate the over-expression of a specific Ca^2+^-permeable channel or the stimulation of a precise cellular process.

Herein, the mechanisms whereby the remodeling of the endothelial transportome, i.e., the specific arsenal of ion channels and transporters expressed by vascular endothelial cells and ECFCs, confers resistance to anti-cancer therapies have been subdivided into two main categories: (1) enhanced neovascularization, which attenuates the therapeutic outcome of anticancer treatments by nourishing cancer cells with O_2_ and nutrients and removing their catabolic waste, and further provides them with a direct access to peripheral circulation, thereby favoring metastasis (Figure 1 and Table 1); and (2) resistance to apoptosis, which hampers the cellular stress induced by chemo- and radiotherapy on tumor endothelial cells and interferes with the dismantling of cancer vasculature (Figure 2 and Table 2).

## 3. Enhanced Neovascularization

### 3.1. Vanilloid Transient Receptor Potential 4 (TRPV4)

TRPV4 has been the first endothelial Ca^2+^-permeable channel to be clearly involved in tumor vascularization [88]. TRPV4 is gated by an array of chemical and physical cues and represents, therefore, the archetypal of polymodal TRP channels [60]. For instance, TRPV4 may be activated by physiological stimuli, including AA and its cytochrome P450-derived metabolites mediators, i.e., EETs, acidic pH, hypotonic swelling, mechanical deformation, heat (>17–24 °C), and dimethylallyl pyrophosphate (DMAPP) [105,106]. Furthermore, TRPV4-mediated Ca^2+^ entry is elicited by manifold synthetic compounds, including the α-phorbol esters, phorbol 12-myristate 13-acetate (PMA) and 4α -phorbol 12,13-didecanoate (4α PDD), and the small molecule drugs, GSK1016790A (GSK) and JNc-440 [60,107]. TRPV4 has long been known to stimulate angiogenesis and arteriogenesis [4,5,108] by stimulating endothelial cell proliferation [5,109] and migration [110]. TRPV4-mediated Ca^2+^ entry is translated into a pro-angiogenic signal by several decoders, such as the Ca^2+^-dependent transcription factors NFAT cytoplasmic 1 (NFATc1), myocyte enhancer factor 2C (MEF2C), and Kv channel interacting protein 3, calsenilin (KCNIP3/CSEN/DREAM), which drive endothelial cell proliferation, [4], β1-integrin and phosphatidylinositol 3-kinase (PI3-K), which promote endothelial cell motility [111]. The opening of only few TRPV4 channels, that tend to assemble into a four-channel cluster, results in spatially-restricted cytosolic Ca^2+^ microdomains, known as Ca^2+^ sparklets, which selectively recruit the downstream Ca^2+^-dependent effectors [112,113]. A recent study revealed that TRPV4 was dramatically up-regulated in breast tumor-derived endothelial cells (B-TECs) and that TRPV4-mediated Ca^2+^ entry significantly increased the rate of cell migration as compared to control cells [88]. TRPV4 promoted B-TEC motility by eliciting local Ca^2+^ pulses at the leading edge of migrating cells [88], which were reminiscent of TRPV4-dependent Ca^2+^ sparklets [112]. TRPV4 was physiologically gated by AA [89], which is quite abundant in breast cancer microenvironment [114]. Likewise, cytosolic phospholipase A2 (PLA2), which cleaves AA from membrane phospholipids in response to physiological stimuli [115] is up-regulated and promotes cancer development by stimulating angiogenesis in several types of tumors, including breast cancer [116]. Therefore, TRPV4 might represent a novel and specific target to treat breast cancer as it is only barely expressed and does not drive migration in healthy endothelial cells [88].

Subsequently, the role of TRPV4 was investigated in prostate adenocarcinoma-derived endothelial cells (A-TECs). Unlike B-TECs, TRPV4 was down-regulated in A-TECs, which increased their sensitivity towards extracellular matrix stiffness, boosted their migration rate and favored the development of an aberrant (i.e., non-uniform, abnormally dilated and leaky) tumor vascular network [90]. This feature gains therapeutic relevance as the resultant hostile (i.e., low extracellular pH, hypoxia, and high interstitial pressure) microenvironment fuels tumor progression and hampers the efficacy of chemotherapy, radiation therapy, anti-angiogenic therapy immunotherapy [117,118]. Accordingly, overexpression or pharmacological activation of TRPV4 with GSK restored A-TEC mechanosensitivity and normalized their abnormal tube formation in vitro by inhibiting enhanced basal Rho activity [91]. Moreover, the daily intraperitoneal injection of GSK was able to normalize tumor vasculature in a xenograft mouse model of Lewis Lung Carcinoma (LLC), thereby improving cisplatin delivery and causing significant tumor shrinkage [91]. In addition, TRPV4-mediated Ca^2+^ entry reduced A-TEC proliferation in vitro by inhibiting the extracellular signal-regulated kinases 1/2 [92]. This mechanism further contributes to GSK-induced dismantling of LLC vasculature in vivo [92]. Therefore, remodeling of TRPV4-mediated Ca^2+^ entry may be used to effectively target tumor vascularization, although the most effective approach may depend on the tumor type. Accordingly, TRPV4 should be inhibited to halt tumor vascularization in breast cancer, while it must be stimulated to normalize tumor vasculature in LLC [23].

### 3.2. Piezo Proteins

Piezo1 and Piezo2 proteins are two recently identified non-selective cation channels that mediate mechanosensory transduction in mammalian cells [119,120]. Piezo proteins are gigantic homotetrameric complexes endowed with one or four ion-conducting pores: each subunit comprises over 2500 amino acids and presents 24–40 predicted transmembrane domains [119]. Piezo channels are Ca^2+^-permeable and, therefore, lead to robust Ca^2+^ entry in response to mechanical deformation of the plasma membrane; unlike TRPV4 channels [105], Piezo-mediated Ca^2+^ entry is directly activated by tension within the lipid bilayer of the plasma membrane rather than by physical coupling to the sub-membranal cytoskeleton or intracellular second messengers [120]. A recent study demonstrated that the endothelial Piezo1 was activated by laminal shear stress to drive embryonic vascular development [81]. Piezo1 promoted vascular endothelial cell migration, alignment and re-alignment along the direction of blood flow by engaging the Ca^2+^-dependent decoders, eNOS and calpain [81,121]. More recently, Piezo2 was found to be up-regulated in T-ECs from mouse xenografted with GL261 glioma cells [93]. Knocking down Piezo2 with a selective small interfering RNA (siRNA) reduced glioma angiogenesis and normalized tumor neovessels [93]. Moreover, suppressing Piezo2 expression decreased VEGF- and interleukin-1β-induced angiogenesis in the mouse corneal neovascularization model [93]. Finally, Piezo2-mediated Ca^2+^ entry elicited the Ca^2+^-dependent transcription of Wnt11 and, consequently, the nuclear translocation of β-catenin in HUVECs, thereby promoting their angiogenic activity in vitro [93]. Although this mechanism remains to be confirmed in T-ECs, Piezo2 stands out as a crucial regulator of tumor angiogenesis and should be probed as a novel target for more effective anti-cancer treatments.

### 3.3. P2X7 Receptors

ATP and its metabolite, adenosine, are major constituents of tumor microenvironment and may differently affect tumor growth, immune cells and tumor-host interaction by activating a wealth of metabotropic (i.e., P2Y1, P2Y2, P2Y4, P2Y6, P2Y11, P2Y12, P2Y13, and P2Y14) and ionotropic (P2X1–2X8,) receptors [122]. Of note, ATP has long been known to stimulate angiogenesis though metabotropic P2y receptors [123]. Nevertheless, a recent investigation demonstrated that P2X7 stimulates tumor angiogenesis in vivo. Two different tumor cell lines, i.e., HEK293 and CT26 colon carcinoma cells, were transfected with P2X7 receptors and subsequently xenografted into immunodeficient or immunocompetent BALB/c mice, respectively. Tumor growth and angiogenesis were significantly enhanced by P_2X7_ expression; consequently, pharmacological inhibition (with AZ10606120) or genetic silencing of P_2X7_ decreased tumor growth and dramatically reduced vascular density [124]. This study further confirmed that P2X7 receptors were significantly up-regulated in several types of cancer cells, including those from breast cancer, and stimulated angiogenesis by promoting VEGF release [124]. More recently, it was found that P2X7 receptors were over-expressed also in B-TECs [94]. This study revealed that the activation of these purinergic receptors with high doses of ATP (>20 μM) and BzATP, a selective P_2X7_ agonist, inhibited B-TEC, but not HDMEC, migration in vitro. The anti-angiogenic effect of P_2X7_ was mediated by the Ca^2+^-sensitive adenylate cyclase 10 (AC10), which increased cyclic adenosine monophosphate (cAMP) and recruited EPAC-1 to dampen cell migration by inducing cytoskeletal remodeling [94]. Moreover, P2X7 receptors-induced cAMP production stabilized bidimensional tumor vessels by favoring pericyte attraction towards B-TECs and reducing endothelial permeability [94]. Intriguingly, hypoxia prevented the anti-angiogenic ability of P_2X7_ receptors by likely reducing their expression [94,125]. These data, therefore, strongly suggest that stimulating P2X7 receptors could provide an efficient strategy to normalize tumor vasculature, thereby enhancing the delivery of cytotoxic drugs and of O_2_ for radiotherapy. In this context, it should be pointed out that P2X7 receptors target hematopoietic EPCs to glioblastoma [126]. Although this investigation was conducted on healthy cells, and remains therefore to be validated in T-EPCs, it suggests that ATP may differentially affect tumor endothelial cells and T-EPCs. Alternatively, the effect exerted by P2X7 con T-EPC fate could be cancer-dependent and needs to be further investigated.

### 3.4. Stim1, Orai1 and Canonical Transient Receptor Potential 1 (TRPC1)

SOCE represents the most important Ca^2+^ entry pathway supporting the pro-angiogenic activity of human ECFCs [29,33,56]. Accordingly, TRPV4 boosted ECFC proliferation rate only when accompanied by the administration of a robust dose of growth factors [86,127], whereas TRPV1 stimulated ECFC proliferation and tubulogenesis by mediating the intracellular intake of anandamide in a Ca^2+^-independent manner [128]. SOCE is activated by the pharmacological (by blocking SERCA-mediated Ca^2+^ sequestration) or physiological (by stimulating InsP_3_Rs) depletion of the ER Ca^2+^ stores and is mediated by the dynamic interplay between Stim1, Orai1 and TRPC1 [29,56,58,84]. It is, however, still unknown whether Orai1 and TRPC1 form two independent Stim1-gated Ca^2+^-permeable routes [129] or assemble into a unique heteromeric supermolecular complex in ECFCs [130]. A recent series of studies revealed that SOCE maintained VEGF-induced intracellular Ca^2+^ oscillations and promoted ECFC proliferation and in vitro tubulogenesis by recruiting the Ca^2+^-dependent transcription factor, NF-κB [58,83]. Of note, SOCE was significantly enhanced in metastatic renal cellular carcinoma (RCC)-derived ECFCs (RCC-ECFCs) due to the up-regulation of Stim1, Orai1 and TRPC1 [95]. Similar to normal cells, the pharmacological blockade of SOCE with YM-58483/BTP2 or with low micromolar doses of lanthanides prevented proliferation and tube formation in RCC-ECFCs [95]. This finding strongly suggests that SOCE could provide an alternative target for the treatment of metastatic RCC [32,131], which develops either intrinsic or acquired refractoriness towards conventional treatments, such as anti-VEGF inhibitors and anti-mammalian target of rapamycin (mTOR) blockers [132]. As more extensively discussed below, the overall remodeling of the intracellular Ca^2+^ toolkit in T-ECFCs could indeed be responsible for the relative or complete failure of standard therapies in RCC patients. Conversely, SOCE was not significantly up-regulated in breast cancer-derived ECFCs (BC-ECFCs) [96]. Accordingly, Orai1 and TRPC1 expression were not significantly altered, while Stim1 was significantly more abundant as compared to control cells. Nevertheless, a tight stoichiometric ratio between Stim1, Orai1 and TRPC1 is required for SOCE to be activated [133]. If all Orai1 and TRPC1 channel proteins are gated by the physiological levels of Stim1, any increase in Stim1 expression will not be sufficient to enhance SOCE as there will be no further channels available on the plasma membrane. Similar to RCC-ECFCs, however, the pharmacological inhibition of SOCE abrogated BC-ECFC proliferation and tube formation, thereby confirming that Orai1 and TRPC1 could serve as reliable targets to interfere with tumor vascularization, although this hypothesis remains to be validated in vivo [134,135]. The strict requirement of Stim1 for tumor vascularization is further suggested by the recent finding that Stim1 transcription in hypoxic tumors is finely regulated by HIF-1 [136]. SOCE, in turn, was found to stimulate HIF-1 accumulation in hypoxic cancer cells by engaging Ca^2+^/calmodulin-dependent protein kinase II and p300 [136]. Therefore, targeting SOCE could also affect the expression of the primary transcription factor responsible for RCC and breast cancer growth and metastasis [137,138]. Of note, HIF-1 has been shown to control also TRPC1 expression [139], although it is still unclear whether this regulation also occurs in tumor microenvironment and, if so, why TRPC1 is up-regulated in RCC-ECFCs, but not in RCC-ECFCs.

The role played by SOCE in tumor vascularization has, finally, been uncovered also in infantile hemangioma (IH), the most common childhood malignancy which may cause disfigurement, ulceration and obstruction and, if not treated, ultimately leads to patients’ death [140]. IH is a vascular tumor that arises as a consequence of dysregulation of angiogenesis and vasculogenesis [140]. The clonal expansion of an endothelial progenitor/stem cell population, which is closely reminiscent of ECFCs, is deeply involved in IH vascularization [141,142]. A recent investigation provided the evidence that Stim1, Orai1 and TRPC1 drive the higher rate of IH-derived ECFC (IH-ECFC) growth as compared to control cells [97]. Stim1, Orai1 and TRPC1 were not up-regulated in IH-ECFCs; however, the ER Ca^2+^ store was depleted to such an extent that Stim1 was basally activated and gated the constitutive activation of Orai1 and TRPC1 [97]. Stim2 displays a lower Ca^2+^ affinity as respect to Stim1 and supports basal Ca^2+^ entry in HUVECs [143]. Nevertheless, the pharmacological abrogation of Stim2 silencing did not affect constitutive SOCE in IH-ECFCs [97]. Constitutive SOCE boosted IH-ECFC proliferation by enhancing NO release [97], thereby emerging as an alternative target to treat IH in propranolol-resistant patients [144].

### 3.5. Neuronal Nicotinic Receptors (nAchRs)

nAchRs belong to a super-family of Cys-loop ligand-gated non-selective cation channels that are physiologically activated by acetylcholine, mediate fast synaptic transmission in neurons and, by virtue of their resolvable Ca^2+^-permeability, control a number of Ca^2+^-dependent processes, including neurotransmitter release and synaptic plasticity [145,146]. However, nAchRs are also largely expressed in non-neuronal brain cells, such as astrocytes, in epithelial cells and in several types of vascular cells, including smooth muscle cells and endothelial cells [75,147,148,149]. It has been established that α7 homomeric nAchRs (α7-nAchRs) promote endothelial cell proliferation, migration and tube formation both in vitro and in vivo by recruiting an array of Ca^2+^-dependent effectors [98,99]. These include eNOS, mitogen-activated protein kinase, phosphoinositide 3-kinase (PI3K), NF-κB, matrix metalloproteinase-2 and -9 [98,99,150]. In addition, α7-nAChRs were shown to induce the JAK2/STAT3 signaling cascade to promote endothelial cell survival [151]. Intriguingly, α7-nAchRs possess the highest Ca^2+^-permeability among the known nAchR subtypes [152]. These pieces of evidence ignited the hypothesis that nicotine accelerated tumor growth by stimulating endothelial α7-nAchRs, thereby promoting angiogenesis and tumor vascularization [147,149]. In support of this model, nicotine induced tumor growth in a mouse model of LLC by stimulating endothelial cell proliferation and tube formation. Nicotine-induced tumor vascularization was significantly reduced by pharmacological blockade (with mecamylamine or hexamethonium) as well as by genetic silencing of α7-nAChRs. The signaling pathways recruited by α7-nAChRs to sustain tumor angiogenesis were not deeply investigated, but nicotine stimulated endothelial cells to release NO, prostacyclin and VEGF [98,99]. It should, however, be pointed out that the expression and role of α7-nAChRs in T-ECs has not been investigated, yet. Nevertheless, hypoxia has been shown to increase α7-nAChRs expression in a mouse model of hindlimb ischemia [98], whereas α7-nAChRs may stimulate HIF-1αtranscription [153]. These observations support the hypothesis that α7-nAChRs are actually over-expressed in T-ECs.

In addition to promoting angiogenesis, nicotine could recruit α7-nAChRs to boost vasculogenesis. A recent study revealed that nicotine induced proliferation, migration and tube formation also in ECFCs and that this effect was inhibited by mecamylamine or α-bungarotoxin [100]. Moreover, nicotine triggered EPC mobilization from bone marrow in a cohort of mice xenografted with colorectal cancer cells, thereby fostering tumor growth and vascularization [101]. Lastly, exposure to second hand smoke stimulated tumor angiogenesis and increased the number of circulating EPCs in a mouse model of LLC by enhancing VEGF release: mecamylamine, however, halted VEGF release, thereby reducing tumor size and capillary density. The pro-angiogenic effect of nicotine was, therefore, likely to be mediated by nAchRs [154]. We are yet to know whether and how α7-nAChRs are altered in T-ECs and T-EPCs. Nevertheless, these ionotropic receptors could be regarded as a promising target for alternative anti-angiogenic therapies.

### 3.6. Gasotransmitters-Activated Ca^2+^-Permeable Channels

Gaseous mediators or gasotransmitters are endogenous signaling messengers that, although being toxic at high concentrations, regulate a multitude of physiological processes, ranging from the regulation of vascular tone to synaptic plasticity and mitochondrial bioenergetics [155,156,157,158]. The gasotransmitters NO and hydrogen sulphide (H_2_S) have recently been shown to stimulate endothelial cells through an increase in [Ca^2+^]_i_ [6,156,159], while the role of CO in angiogenesis is less clear [160]. NO promotes angiogenesis and disease progression in several types of malignancies [161,162], including breast cancer [163]. The administration of two structurally unrelated NO donors, i.e., S-nitroso-*N*-acetylpenicillamine (SNAP) or sodium nitroprusside (SNP), was recently found to trigger Ca^2+^ influx and migration in B-TECs [164]. These effects were mimicked by elevating endogenous NO release with l-arginine [164], which is the physiological substrate for eNOS [156]. Of note, AA-induced TRPV4 activation in B-TECs was inhibited by preventing NO production with *N* ^G^-nitro-l-arginine methyl ester (l-NAME) [88,164]; moreover, AA- and NO-induced Ca^2+^ entry were both sensitive to protein kinase A (PKA) inhibition [164]. It is, therefore, likely that NO elicits Ca^2+^ entry in B-TECs by gating TRPV4. In agreement with this hypothesis, TRPV4 may be activated by NO through direct S-nitrosylation [71] and is phosphorylated by PKA upon AA stimulation in vascular endothelial cells [70]. Finally, NO-induced Ca^2+^ entry and migration were dramatically reduced in HDMECs [164], in which TRPV4 expression was significantly down-regulated [88]. Besides TRPV4, however, NO is able to recruit multiple TRP channels, such as TRPC1, TRPC4, TRPC5, TRPV1, and TRPV3 [71], some of which are up-regulated in T-ECFCs [95,165]. Unfortunately, it is still unclear whether NO elicits intracellular Ca^2+^ entry in these cells. Although future work is mandatory to understand whether NO stimulates TRP channels, as well as other Ca^2+^-permeable channels, to promote tumor vascularization, endothelial Ca^2+^ signaling is emerging as an attractive target to prevent its pro-tumorigenic effect.

H_2_S has also been shown to promote angiogenesis in a Ca^2+^-dependent manner. For instance, H_2_S mediated VEGF-induced Ea.hy926 cell proliferation and migration by inducing InsP_3_-dependent ER Ca^2+^ release without the contribution of extracellular Ca^2+^ entry [6]. The components of the endothelial Ca^2+^ toolkit recruited by H_2_S may, however, vary depending on the vascular bed [166]. H_2_S induced ER-dependent Ca^2+^ release through InsP_3_Rs and RyRs followed by a sustained SOCE in primary cultures of human saphenous vein endothelial cells [167], whereas it recruited the reverse mode of NCX by gating a Na^+^- and Ca^2+^-permeable pathway in rat aortic endothelial cells [168] and HDMECs [21]. Conversely, NaHS did not elicit any resolvable elevation in [Ca^2+^]_i_ in ECFCs [6] and its role in neovasculogenesis in vivo operated by truly endothelial precursors remains to be elucidated [169]. H_2_S-induced Ca^2+^ signals may be translated into a pro-angiogenic signal by multiple Ca^2+^-dependent decoders, including the PI3K/Akt and the ERK/p38 signaling pathways [155,156]. Growing evidence demonstrated that H_2_S drove disease progression and angiogenesis in several types of tumor, such as RCC and colorectal cancer [169]. Intriguingly, sodium hydrosulfide (NaHS), a widely employed H_2_S donor, induced intracellular Ca^2+^ signals in both B-TECs and HDMECs; however, NaHS-elicited Ca^2+^ signals were enhanced and arose within a significantly lower range (nanomolar vs. micromolar) in B-TECs [21]. Consequently, NaHS promoted proliferation and migration in B-TECs, but not in control endothelial cells [21]. The Ca^2+^ response to H_2_S was mediated by a Ca^2+^-permeable non-selective cation channel and was sustained by membrane hyperpolarization through the activation of a K^+^ conductance [21], likely an ATP-sensitive K^+^ channel [157]. The molecular nature of this Ca^2+^-permeable route is yet to be identified [166]. Nevertheless, H_2_S is able to stimulate TRPV3 and TRPV6 in bone marrow-derived mesenchymal cells by direct sulfhydration of some of Cys residues within their protein structure [170]. Moreover, H_2_S activated TRPA1 in RIN14B cells [171]. Deciphering the molecular target of H_2_S in tumor endothelium is, therefore, mandatory to devise alternative anti-cancer treatments. In addition, both eNOS and cys-tathionine gamma lyase (CSE), the enzyme which catalyzes H_2_S production in vascular endothelial cells, are Ca^2+^-sensitive [16,42,172]. Therefore, targeting a Ca^2+^ entry/release pathway tightly coupled to either eNOS (i.e., Orai1, [173]) or CSE (yet to be identified) has the potential to interfere with multiple pro-angiogenic pathways and, therefore, exert a more profound anti-tumor effect. 

### 3.7. Connexin 40 (Cx40)

Connexin (Cx) hemichannels, also termed connexons, have long been known to provide the building blocks of gap junctions, thereby enabling the transfer of small solutes, ions and signaling molecules, such as Ca^2+^ and InsP_3_, between adjacent cells [174]. Three diverse Cx isoform exist in vascular endothelial cells, i.e., Cx37, Cx40, and Cx43, and synchronize robust NO release induced by extracellular autacoids by mediating intercellular Ca^2+^ communication [175,176]. In addition, unopposed Cx hemichannels were found to mediate extracellular Ca^2+^ entry and NO release in endothelial cells from different vascular beds [16,74,177,178,179]. Earlier work suggested that Cxs served as tumor suppressors and were down-regulated in cancer, thereby affecting vascular integrity and reducing vascular leakage [180,181,182]. However, a recent study challenged this dogma by showing that Cx40 was over-expressed in T-ECs and promoted disease progression and angiogenesis in melanoma and urogenital cancers [102]. Cx40 stimulated tumor growth by inducing eNOS recruitment, which strongly suggest that intracellular Ca^2+^ levels increased during the angiogenic process [102]. Intriguingly, targeting Cx40 function with ^40^Gap27, a peptide that has long been use to inhibit Cx40-mediated intercellular communication and extracellular Ca^2+^ entry [1,16,178], normalized tumor vasculature and enhanced the efficacy of the chemotherapeutic drug, cyclophosphamide [102]. Therefore, although these findings remain to be confirmed in other tumor types, and the role served by Ca^2+^ is still unclear, Cx40 deserves careful consideration for the design of new anticancer drugs.

### 3.8. Na^+^/H^+^ Exchanger-1 (NHE-1)

The Na^+^/H^+^ exchanger NHE-1 is a reversible electroneutral antiporter that maintains cytosolic pH by expelling H^+^ at expense of the inwardly directed Na^+^ electrochemical gradient with a 1:1 stoichiometric ratio [183]. NHE-1 induces endothelial cell proliferation, migration and tube formation by means of several Ca^2+^-dependent effectors, such as calpain [184], eNOS [185], and ERK 1/2 [186]. Accordingly, thrombin-induced NHE-1 activation was able to increase sub-membranal Na^+^ levels, thereby switching NCX into the reverse mode and mediating extracellular Ca^2+^ entry in HUVECs [187,188]. Moreover, NHE-1-induced cytosolic alkalinization triggered ER-dependent Ca^2+^ release through InsP_3_Rs in bovine aortic endothelial cells and human pulmonary artery endothelial cells [189,190]. NHE1 is constitutively activated in cancer cells to favor extracellular acidification and stimulate metastasis and invasion by facilitating protease-mediated degradation of the extracellular matrix [191]. In addition, NHE-1 is transcriptionally regulated by HIF-1 and is up-regulated in a multitude of carcinomas [191,192]. A recent series of studies demonstrated that NHE-1 was over-expressed in endothelial cells exposed to tumor microenvironment [193] and was able to boost vascularization, invasion and metastasis in several types of tumors, including breast cancer [191,194]. Accordingly, aldosterone-induced NHE-1 activation promoted B-TEC proliferation, migration and cytosolic alkalinization [103]. Further work is required to assess whether NHE-1 activation stimulates tumor vascularization through an increase in [Ca^2+^]_i_. However, NHE-1 blockers, including cariporide and the more specific 3-methyl-4-flouro analog of 5-aryl-4(4-(5-methyl-14-imidazol-4-yl) piperidin-1-yl)pyrimidine (Compound 9t), have been put forward as alternative anti-cancer drugs [195].

### 3.9. Two-Pore Channels (TPCs)

The ER is the largest endogenous Ca^2+^ store in vascular endothelial cells by accounting for ≈75% of the total Ca^2+^ storage capacity [2]. The remainder 25% of the total stored Ca^2+^ is located within the mitochondria and the acidic Ca^2+^ stores of the endolysosomal (EL) system [2]. As more widely illustrated in [196], the EL Ca^2+^ store releases Ca^2+^ through many Ca^2+^-permeable channels, including Mucolipin TRP 1 (TRPML1), Melastatin TRPM 2 (TRPM2) and two-pore channels 1 and 2 (TPC1–2) [197,198]. The newly discovered second messenger, nicotinic acid adenine dinucleotide phosphate (NAADP), is the physiological stimulus that gates TPC1–2 in response to extracellular stimulation [196,199,200]. NAADP-induced EL Ca^2+^ release is, in turn, amplified by juxtaposed ER-embedded InsP_3_Rs and RyRs through the CICR process, thereby initiating a regenerative Ca^2+^ wave [196,200,201]. NAADP-gated TPC2 channels are also expressed in vascular ECs [202,203], whereas *N-*ECFCs display larger amounts of TPC1 [86,204]. A recent study demonstrated that NAADP-induced Ca^2+^ signals promoted tumor vascularization and metastasis in murine models xenografted with B16 melanoma cells [205]. Of note, the pharmacological blockade of NAADP-induced Ca^2+^ release with Ned-19 dampened melanoma growth, vascularization and lung metastasis [205]. Future work will have to assess whether TPC2 channels are up-regulated in T-ECs and whether NAADP-induced Ca^2+^ signaling also drive T-ECFC incorporation into tumor neovessels. However, TPCs stand out as promising targets to develop alternative anti-angiogenic treatments.

## 4. Resistance to Apoptosis

### 4.1. Canonical Transient Receptor Potential 5 (TRPC)

TRPC5 forms a homotetrameric Ca^2+^-permeable channel that is gated upon PLCβ activation by Gq/11-coupled membrane receptors through a yet to be identified signaling cascade [2,206,207]. Accordingly, although some studies reported that TRPC5 is recruited in a store-dependent manner by Stim1 [208], it has been proposed that TRPC5 activation by PLCβ does not involve ER store depletion [209]. In addition, TRPC5-mediated Ca^2+^ entry is elicited by several physiological messengers, including reduced thioredoxin, protons, sphingosine-1-phosphate, lysophospholipids, NO and Ca^2+^ itself [207]. Finally, TRPC5 presents a spontaneous activity that is increased by lanthanides, cold temperatures (47 °C to 25 °C) and membrane stretch; consequently, TRPC5 serves as a cold sensor in the peripheral nervous system [210]. Of note, TRPC5 may establish physical associations with a multitude of molecular partners, including TRPC1, TRPC4 and TRPC6, which regulate its membrane localization and biophysical properties [207]. TRPC5 differently tunes angiogenesis depending on the vascular bed. For instance, TRPC5 promoted proliferation and tube formation by inducing intracellular Ca^2+^ oscillations in EA.hy926 cells [211]. Conversely, a TRPC6-TRPC5 channel interaction inhibited angiogenesis by decreasing the rate of migration in bovine aortic ECs (BOECs). In this context, TRPC6-mediated Ca^2+^ entry triggered an ERK-mediated phosphorylation cascade that leads to MLCK activation and TRPC5 externalization on the plasma membrane [212,213]. It has recently been shown that endothelial TRPC5 could underlie the development of chemoresistance to anticancer drugs in both breast cancer [104,214,215] and colorectal carcinoma [216]. P-glycoprotein (P-gp), also termed multidrug resistance protein 1 (MDR1), is a multidrug efflux transporter that expels xenobiotics out from the cytoplasm into the extracellular milieu [217]. P-gp overexpression, therefore, confers resistance to malignant cells, which become insensitive to a wide range of cancer chemotherapeutics, including adriamycin, vincristine, taxol, and anthracyclines [217]. Earlier evidence demonstrated that TRPC5 was up-regulated and induced P-gp overexpression by hyper-stimulating the Ca^2+^-dependent transcription factor, NFATc3 in chemoresistant MCF-7 breast cancer cells [214]. In agreement with this observation, microRNA 320a (miR-320a), which is able to associate with and degrade TRPC5 and NFATc3 transcripts in normal cells, was down-regulated in chemoresistant breast cancer cells due to the hypermethylation of its promoter sequence [218]. TRPC5 up-regulation induced resistance to adriamycin, paclixatel, epirubicin, mitoxantrone and vincristine [214]. Additionally, TRPC5-mediated Ca^2+^ entry promoted transcription of HIF-1α gene, thereby boosting VEGF release and enhancing tumor angiogenesis [219]. Remarkably, TRPC5-based chemoresistance could be shuttled to tumor endothelial via intercellular communication. Adriamycin-resistant MCF-7 cells could pack the up-regulated TRPC5 channels into mobile extracellular vesicles (EVs), which are released in tumor microenvironment and transferred their signaling content to surrounding endothelial cells. This scenario is supported by the observations that HDMECs exposed to TRPC5-contanining EVs, which were collected from adriamycin MCF-7 breast cancer cells, over-expressed the TRPC3-NFATc3-P-gp signaling pathway and developed resistance to adriamycin-induced apoptosis [104]. Moreover, TRPC5-containing vesicles were identified in peripheral blood of breast cancer patients receiving chemotherapy and of nude mice bearing adriamycin-resistant MCF-7 tumor xenografts [215]. Furthermore, P-gp production was enriched in tumor endothelium of adriamycin-resistant MCF-7 xenografts than in other sites and was sensitive to TRPC5 inhibition with a specific siRNA (siTRPC5) [104]. These data, therefore, suggest that TRPC5 provide a promising target to design alternative adjuvant anticancer treatments [220]. Accordingly, a blocking TRPC5 antibody reduced P-gp expression, retarded cancer growth and boosted paclitaxel-induced tumor regression in chemoresistant breast cancer in vivo [104,214,215]. The endothelial effects of TRPC5 in breast cancer are seemingly limited to T-ECs, as BC-ECFCs do not express this channel [96]. Future work will have to assess whether, besides conferring B-TECs with the resistance to chemotherapeutic drugs, TRPC5 up-regulation accelerates breast cancer angiogenesis. 

### 4.2. Inositol-1,4,5-Trisphosphate (InsP_3_) Receptors (InsP_3_Rs)

**Figure 3:**
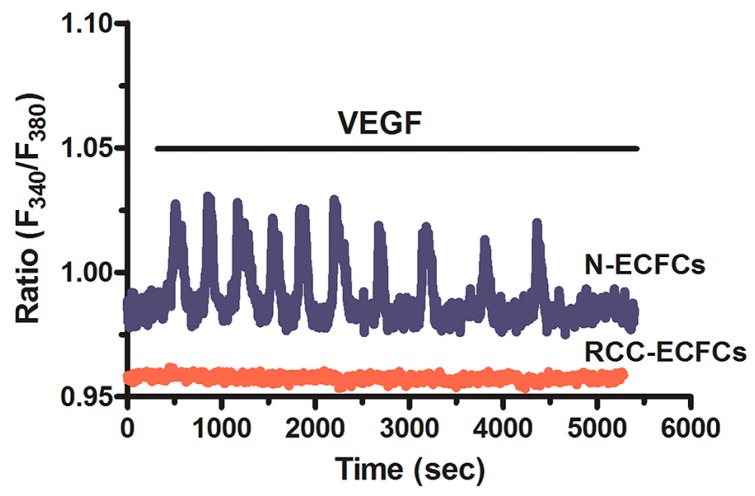
VEGF does not trigger pro-angiogenic Ca^2+^ oscillations in tumor-derived endothelial colony forming cells. VEGF (10 ng/mL) triggers intracellular Ca^2+^ oscillations in *N-*ECFCs, but not in RCC-ECFCs. Adapted from [95].

InsP_3_Rs are non-selective cation channels which mediate ER-dependent Ca^2+^ release, thereby controlling multiple endothelial cell functions, including bioenergetics, apoptosis, angiogenesis and vasculogenesis (see Paragraph 2. Ca^2+^ signaling in normal endothelial cells: a brief introduction). Three distinct InsP_3_R isoforms exist in both vascular endothelial cells and ECFCs [2,29], i.e., InsP_3_R1, InsP_3_R2 and InsP_3_R3, which may associate into homo- or hetero-tetrameric ER-embedded channels [2]. It has recently been shown that InsP_3_Rs were dramatically down-regulated in RCC-ECFCs, thereby preventing the onset of VEGF-induced intracellular Ca^2+^ oscillations, proliferation and in vitro tubulogenesis [95] (Figure 3). More specifically, RCC-ECFCs only expressed InsP_3_R1, while InsP_3_R2 and InsP_3_R3 were absent [95]. This result was surprising as InsP_3_R1 was transcriptionally regulated by HIF-2 in human RCC cancer cell lines [221]. The failure of the pro-angiogenic Ca^2+^ response to VEGF also involves the chronic reduction in the ER Ca^2+^ concentration ([Ca^2+^]_ER_) in RCC-ECFCs, as monitored by using an ER-targeted aequorin Ca^2+^ indicator [87,222]. Therefore, in contrast with the widely accepted belief that VEGF sustains the angiogenic switch [223], VEGF does not stimulate ECFC-dependent neovessel formation in RCC patients [27,32]. This observation shed novel light on the refractoriness to anti-VEGF therapies in individuals suffering from RCC [224,225,226]. It has long been known that humanized monoclonal anti-VEGF antibodies, such as bevacizumab, or small molecule tyrosine kinase inhibitors, such as sorafenib and sunitinib, did not increase the overall survival of RCC patients, who ultimately developed secondary (acquired) resistance and succumbed because of tumor relapse and metastasis. In addition, targeting VEGF-dependent pathway proved to be ineffective in a large cohort of subjects, who displayed intrinsic refractoriness to these anti-VEGF drugs and did not show any improvement in their progression free survival [227,228]. ECFCs play a key role during the early phases of the angiogenic switch that supports tumor vascularization and metastasis [27,28,229]. If tumor vasculature is dismantled by anti-VEGF drugs, the following drop in P_O2_ will release in circulation a cytokine storm that attracts ECFCs from their bone marrow and/or vascular niches. ECFCs will home to the shrunk tumor, but, being insensitive to VEGF, will not be affected by the presence of anti-VEGF drugs. Consequently, they will proliferate in response to the mixture of growth factors liberated in tumor microenvironment and will restore blood supply to cancer cells [32]. Remodeling of the Ca^2+^ toolkit in RCC-ECFCs could, therefore, underlie the resistance to anti-angiogenic therapies in RCC patients. Similar data were obtained in BC-ECFCs, in which the significant reduction in [Ca^2+^]_ER_ prevented VEGF from triggering robust intracellular Ca^2+^ oscillations, proliferation and tube formation, although the pattern of InsP_3_R expression remained unchanged [37,96]. Again, this result is consistent with notion that also breast cancer patients present intrinsic or secondary refractoriness to anti-VEGF therapies [54,230]. The reduction in [Ca^2+^]_ER_ observed in several types of tumor-associated ECFCs, including IH-ECFCs [95,96,97], was likely to reflect the down-regulation of SERCA2B activity [87,95]. Accordingly, ATP-induced InsP_3_-dependent ER Ca^2+^ release in RCC-ECFCs decayed to resting Ca^2+^ levels with slower kinetics as compared to normal ECFCs [95]. Intriguingly, the gene expression profile of RCC- and BC-ECFCs resulted to be dramatically different with respect to normal cells [37]: BC-ECFCs and RCC-ECFCs presented, respectively, 382 and 71 differently expressed genes (DEGs) as compared to healthy cells, including TMTC1 [37]. TMTC1 is a tetratricopeptide repeat-containing adapter protein, which binds to and inhibits SERCA2B, thereby reducing ER Ca^2+^ levels and dampening agonist-induced intracellular Ca^2+^ release [231]. It is conceivable that TMTC1 up-regulation in T-ECFCs contributes to the chronic underfilling of their ER Ca^2+^ reservoir. In further agreement with this observation, electron microscopy revealed that both RCC- and BC-ECFCs presented dramatic ultrastructural differences as compared to control cells [87,96]. In particular, T-ECFCs presented a remarkable expansion of ER volume, whereas mitochondria were more abundant and very often elongated as compared to *N-*ECFCs [87,96]. This ultrastructural remodeling is consistent with the ER stress caused by the chronic reduction in [Ca^2+^]_ER_ [232,233].

## 5. Targeting the Endothelial Ca^2+^ Toolkit to Circumvent the Resistance to Anticancer Treatments

Remodeling of the Ca^2+^ toolkit in tumor cells led many authors to search for alternative strategies to treat cancer. Intracellular Ca^2+^ signaling controls most, if not all, the so-called cancer hallmarks and could, therefore, be targeted to inhibit or, at least, retard tumor growth and metastasis [35,36,234,235,236,237,238,239,240]. As shown above, the Ca^2+^ transportome is also altered in stromal cancer cells [32,241,242], including endothelial cells and ECFCs. Remodeling of the endothelial Ca^2+^ toolkit could play a crucial role in the refractoriness to anticancer treatments, by supporting tumor vascularization and decreasing the susceptibility to pro-apoptotic stimuli. Therefore, the endothelial Ca^2+^ transportome might provide an efficient target for adjuvant therapies to conventional anti-cancer treatments. Three strategies could be pursued to improve the therapeutic outcome of standard therapies by interfering with the endothelial Ca^2+^ machinery: (1) blocking Ca^2+^ signaling to dampen angiogenesis and/or vasculogenesis; (2) stimulating Ca^2+^ entry to normalize tumor vessels, thereby improving the delivery and efficacy of chemo-, radio- and immunotherapy; and (3) manipulating Ca^2+^ signaling to endothelial cell apoptosis and dismantle tumor vasculature.

SOCE is, perhaps, the most suitable target to affect tumor vasculature by inhibiting both angiogenesis and vasculogenesis. Although there is no report of SOCE expression in T-ECs, Stim1 and Orai1 control proliferation and tube formation in normal endothelial cells, such as rat CMECs [7], bovine brain capillary endothelial cells [243], mouse lymphatic endothelial cells [244], and HUVEC [57,58]. Moreover, the pharmacological blockade of SOCE attenuates the rate of cell growth and abrogates in vitro tubulogenesis in RCC-, IH- and BC-derived ECFCs [95,96,97]. In addition, SOCE controls proliferation, migration and metastasis in a multitude of different cancer cell lines [134,135], which expands the cellular targets of SOCE inhibitors to the whole tumor microenvironment. We [131,135] and others [133,245] have recently described the Orai1 and TRPC1 inhibitors, some of which have been listed in Table 1, that could serve as a molecular template to design novel anticancer drugs. Unfortunately, none of these drugs have reached the milestone of being approved by US Food and Drug Administration (FDA) due to their scarce selectivity and high toxicity. For instance, carboxyamidotriazole (CAI), a non-selective blocker of Ca^2+^ signaling, was originally used to inhibit angiogenesis in vitro and tumor vascularization in vivo [131,246,247,248]. Depending on the cell type, CAI was able to block SOCE by occluding the mitochondrial Ca^2+^ uniporter [249,250] or reducing InsP_3_ synthesis, which in turn prevents InsP_3_-dependent Ca^2+^ release and Stim activation [56,251,252]. Intriguingly, CAI inhibited proliferation and tube formation also in RCC- and BC-ECFCs by preventing InsP_3_-dependent ER Ca^2+^ release [95,96]. Additionally, CAI was found to block growth and motility in several types of cancer cell lines [252,253]. Therefore, phase I-III clinical trials were launched to assess CAI toxicity and tolerability in patients suffering from several types of malignancies, including RCC, breast cancer, ovarian cancer, melanoma, non-small cell lung carcinoma, and gastrointestinal (stomach and pancreas) adenocarcinomas [56,248,254,255]. As discussed elsewhere [131], this drug caused disease stabilization when administrated alone or as adjuvant of chemo- or radio-therapy, and induced well tolerable side effects in most patients, such diarrhea, nausea and/or vomiting, fatigue and constipation. The therapy was discontinued only in RCC patients, who underwent disease progression and suffered from unacceptable toxicities, such as neuropsychiatric difficulties and asthenia [256]. As mentioned earlier, however, the effect of CAI is not directed towards the SOCE machinery, but is indirect. In addition to SOCE, CAI may also block TRPV4 and ER leakage channels [89,95,96,131]. A recent investigation, however, screened a library of >1800 FDA-approved drugs to search for specific SOCE blockers and identified five novel compounds, i.e., leflunomide, teriflunomide, lansoprazole, tolvaptan and roflumilast, that could be successfully used in therapy (leflunomide and terifluonomide) or provide the template to design more selective Orai1 inhibitors (i.e., lansoprazole, tolvaptan and roflumilast) [257].

An alternative strategy consists in stimulating endothelial Ca^2+^ signaling to induce tumor normalization by activating distinct Ca^2+^ entry routes depending on the tumor type. For instance, TRPV4-mediated Ca^2+^ entry drives tumor normalization in LLC [90,91], whereas P_2X7_ receptors could be targeted to normalize tumor vasculature in breast cancer [94]. Tumor normalization, in turn, represents a promising adjuvant approach to facilitate cancer therapy by increasing the diffusion of chemotherapeutic drugs, improving radiotherapy efficiency and favoring the recruitment of tumor-killing immune cells [118,258]. Several synthetic agonists may selectively induce TRPV4 opening, such as 4αPDD derivatives, RN-1747, and JNc-440. Moreover, GlaxoSmithKline commercialized several patent applications of small molecule TRPV4 activators, the most famous of which is GSK [105,259]. Likewise, BzATP is regarded as the most potent P_2X7_ receptor agonist, while 2-meSATP and ATPγS are only partial agonists and αβ-meATP and βγ-meATP exert a rather weaker on activation [260]. Clearly, further studies are required to uncover additional components of the endothelial Ca^2+^ toolkit potentially implicated in tumor normalization. Nevertheless, a recent investigation reported that angiopoietins, which induce vessel maturation by regulating the interaction between luminal endothelial cells and mural cells, such as vascular smooth muscle cells and pericytes, stimulate HUVEC migration by promoting ER-dependent Ca^2+^ release through InsP_3_Rs and RyRs [261]. These findings lend further support to the hypothesis that targeting the endothelial Ca^2+^ signaling provides a suitable means to accelerate the dismantling of tumor vasculature by standard anticancer therapies.

Finally, the endothelial Ca^2+^ machinery could be properly manipulated to enhance the pro-apoptotic outcome of chemo- and radiation-therapy. For instance, TRPC5-mediated Ca^2+^ entry could be inhibited in B-TECs by taking advantage of a battery of novel small molecule inhibitors, such as Pico145 [262], 3,5,7-trihydroxy-2-(2-bromophenyl)-4H-chromen-4-one (AM12) [263], 2-aminobenzimidazole derivatives [264], ML204 [265], and neuroactive steroids [266]. Alternatively, the [Ca^2+^]_ER_ could be augmented to such an extent to induce the pro-apoptotic InsP_3_-driven ER-to-mitochondria Ca^2+^ transfer. Pinton’s group demonstrated that phototherapy induces a p53-dependent increase in [Ca^2+^]_mit_, which leads to tumor disruption in vivo [239,267]. Moreover, cytotoxic ER-dependent Ca^2+^ mobilization could be promoted by conjugating thapsigargin, a selective SERCA inhibitor, with a protease-specific peptide carrier, which is cleaved by the prostate-specific membrane antigen (PMSA) [268]. PMSA is widely expressed in the endothelium of many solid tumors [269,270], including RCC, thereby selectively favoring thapsigargin release in TME and inducing cancer and stromal cell apoptosis [268,271]. This prodrug has been termed mipsagargin or prodrug G202 and has recently been probed in a phase I clinical trials in patients suffering from refractory, advanced or metastatic solid tumors [272]. We do not know yet whether [Ca^2+^]_ER_ is also decreased in the endothelium of tumor neovessels, as ECFCs are likely to be replaced/diluted by local endothelial cells after the angiogenic switch [27]. Nevertheless, mipsagargin is likely to cause pro-apoptotic Ca^2+^ release in all stromal cells, including T-ECs.

As outlined elsewhere [23,32,135], caution is warranted when targeting a ubiquitous intracellular second messenger, such as Ca^2+^. It should, however, be pointed out that several inhibitors of voltage-gated Ca^2+^ channels, such as verapamil, nifedipine and nitrendipin, are routinely employed in clinical practice to treat severe cardiovascular disorders, including hypertension, arrhythmia, acute myocardial infarction-induced heart failure and chronic stable angina [135,273]. In agreement with this observation, a phase I clinical trial is currently assessing the therapeutic outcome of Ca^2+^ electroporation on cutaneous metastases of solid tumors as compared to standard electrochemotherapy with bleomycin (https://clinicaltrials.gov/ct2/show/NCT01941901). Ca^2+^ electroporation is predicted to enhance the rate of cancer cell death by resulting in cytotoxic Ca^2+^ accumulation in the cytosol and in mitochondria [36]. Finally, the pharmacological inhibition of intracellular Ca^2+^ signaling did not elicit any intolerable side effects, such as immune depression, bleeding or neuropathic disorders, in least three distinct models of human cancer xenografts [24,205,274].

## 6. Conclusions

The present article discussed how remodeling of the endothelial Ca^2+^ toolkit (or transportome) could contribute to the resistance to anti-cancer treatments, which hampers from the very beginning their therapeutic outcome (intrinsic resistance) or leads to tumor relapse (acquired resistance) and patients’ death. The intimate relationship between endothelial Ca^2+^ signaling and refractoriness to anti-cancer treatments cannot be fully appreciated by studying normal/healthy endothelial cells [32]. For instance, the role of VEGF in promoting tumor neovascularization has been extensively acknowledged based upon the observation that VEGF triggers pro-angiogenic Ca^2+^ signals in normal endothelial cells [6,58,251] and ECFCs. Nevertheless, VEGF does not stimulate proliferation and tube formation in T-ECFCs, which play a crucial role in sustaining the angiogenic switch and are likely to restore tumor vasculature prior to recurrence of disease progression. Therefore, to be effective in the patients, a strategy aiming at targeting the Ca^2+^ toolkit must be first probed on tumor-associated endothelial cells and ECFCs in vitro, as the their Ca^2+^ machinery could be different from that of naïve cells. The protocol to isolate ECFCs from peripheral blood does not require an unreasonable volume of blood (≈40 mL), but it takes no less than three weeks [31] due to lack of ECFCs-specific membrane antigens. It will be imperative to speed up this procedure to accelerate the therapeutic translation of the findings generated by basic research. Isolating T-ECs represents a more technically demanding challenge, but several strategies were designed to collect and expand T-ECs from several types of solid cancers [275]. Further work on patients-derived T-ECs or T-ECFCs is mandatory to identify novel components of the endothelial Ca^2+^ toolkit involved in the refractoriness to anti-cancer therapies. Most of the attention is, of course, currently paid to Stim1 and Orai1 [33,131] and to the multiple TRP channel subfamilies that drive physiological angiogenesis [2,131,276,277]. Additional components of the endothelial Ca^2+^ transportome deserve careful investigation. For instance, Orai3 was found to up-regulated in several types of T-ECFCs [95,165], but its role in tumor vascularization is currently unknown. Of note, Orai3 may replace Orai1 as the pore-forming subunit of store-operated channels in cancer cells and could, therefore, emerge as a promising target for anti-cancer therapies [278]. NCX provides another unconventional Ca^2+^ entry that regulates proliferation and tube formation in healthy endothelial cells [18,279], but has been scarcely investigated in tumor neovessels. Finally, nAchRs are not the only ionotropic receptors expressed in vascular endothelial cells. *N-*methyl-d-aspartate receptors (NMDARs) are widely expressed in brain microvascular endothelial cells [280], in which they recruit eNOS and stimulate NO release in response to synaptic activity [281]. Aberrant glutamate signaling has been associated to glioma growth [282] and NMDARs-mediated Ca^2+^ entry could engage Ca^2+^-dependent decoders other than eNOS in brain endothelium. Therefore, the expression and role of endothelial NMDARs in glioblastoma should be carefully evaluated.

## Figures and Tables

**Table 1:** Channels and transporters directly supporting tumor vascularization.

Channel/Transporter	Tumor and Cell Type (T-EC, T-ECFC T-EPC)	Expression Levels (Transcripts and/or Proteins)	Effect on Tumor Vascularization	Strategy to Target Tumor Vascularization	Ref.
TRPV4	Breast Cancer: T-ECs	↑	Stimulates B-TEC proliferation, migration and in vitro tubulogenesis	Channel blockade with shTRPV4 or with CAI (0.1–10 µM)	[88,89]
TRPV4	Lewis Lung Carcinoma: T-ECs (isolated from prostate adenocarcinoma)	↓	Inhibits T-EC mechanosensation, proliferation and migration in vitro and promotes the formation of a malfunctioning, leaky and exceedingly expanded vascular network in vivo	Injection of TRPV4 agonist GSK (10 μg/kg) to normalize tumor vasculature and favor cisplatin-induced tumor regression	[90,91,92]
Piezo2 proteins	Glioma: T-ECs	↑	Regulates tumor angiogenesis, vascular leakage and permeability	Blockade with siPiezo2	[93]
P2X7Rs	Breast cancer: T-ECs	↑	Inhibits B-TEC migration and normalizes B-TECs-derived vessels in vitro	Activated by BzATP (50 µM)	[94]
Stim1, Orai1, TRPC1	Renal cellular carcinoma: T-ECFCs	↑	Stimulate T-EPC proliferation and in vitro tubulogenesis	Blockade with siStim1 and siOrai1 and with YM-58483/BTP2 (20 µM), La^3+^ (10 µM), Gd^3+^ (10 µM), CAI (2–10 µM), 2-APB (50 µM), and genistein (50 µM)	[95]
Stim1, Orai1, TRPC1	Breast cancer: T-ECFCs	=	Control T-ECFC proliferation and in vitro tubulogenesis	Blockade with YM-58483/BTP2 (20 µM), La^3+^ (10 µM), and CAI (10 µM)	[96]
Stim1, Orai1, TRPC1	Infantile hemangioma: T-ECFCs	↑	Control T-ECFCs proliferation in vitro	Blockade with with YM-58483/BTP2 (20 µM), La^3+^ (10 µM), and Pyr6 (10 µM)	[97]
α7-nAchRs	Lewis lung carcinoma: T-ECs and T-EPCs	Not determined	Controls tumor growth and angiogenesis in vivo	Blockade with mecamylamine (1.0 μg/kg) or hexamethonium (1.0 μg/kg)	[98,99]
			Stimulates EPC proliferation, migration and tubulogenesis in vitro and EPC recruitment in vivo	Blockade in vitro with mecamylamine (1 µM) and α-bungarotoxin (10 nM) and in vivo with mecamylamine (0.24 mg/kg per day)	[100,101]
Connexin40	Melanoma and urogenital cancers: T-EC	↑	Stimulates tumor angiogenesis and growth in vivo	Blockade in vivo with ^40^Gap^27^ peptide (100 μg)	[102]
NHE-1	Breast cancer: TECs	Not determined	Stimulates B-TEC migration in vitro	Blocked with siNHE-1 and with cariporide (50 µM)	[103]

The generic term EPC, in this context, refers to circulating pro-angiogenic cells which cannot be grouped into the ECFC sub-family and are likely to belong to the myeloid lineage.

**Table 2:** Components of the endothelial Ca^2+^ toolkit that determine endothelial cell resistance to chemotherapeutic drugs.

Channel/Transporter	Tumor and Cell Type (T-EC and T-EPC)	Expression Levels	Effect on Tumor Vascularization	Strategy to Target Tumor Vascularization	Ref.
TRPC5	Breast Cancer: T-ECs	↑	Stimulates endothelial resistance to adriamycin	Channel blockade with the specific blocking antibody T5E3 (concentration not reported)	[104]
InsP_3_Rs	RCC: T-ECFCs	↓	Favor T-ECFC resistance to rapamycin	Preventing InsP_3_-dependent ER–mitochondria Ca^2+^ shuttle with selective InsP_3_R inhibitors or cytosolic Ca^2+^ buffers (e.g., BAPTA)	[87]
